# Morphologic characteristics of severe basilar artery atherosclerotic stenosis on 3D high-resolution MRI

**DOI:** 10.1186/s12883-018-1214-1

**Published:** 2018-12-15

**Authors:** Runcai Guo, Xuebin Zhang, Xianjin Zhu, Zunjing Liu, Sheng Xie

**Affiliations:** 1 Department of Radiology, China-Japan Friendship Hospital, 2 Yinghuayuan Dongjie, Beijing, China; 2 Department of Neurology, China-Japan Friendship Hospital, 2 Yinghuayuan Dongjie, Beijing, China

**Keywords:** Basilar artery, High-resolution magnetic resonance imaging, Remodeling index, Percutaneous transluminal angioplasty and stenting, Volumetric isotropic turbo spin echo acquisition

## Abstract

**Background:**

Two-dimensional high-resolution MRI (2D HRMRI) faces many technical challenges for fully assessing morphologic characteristics of inherent tortuous basilar arteries. Our aim was to investigate remodeling mechanisms and plaque distribution in symptomatic patients with basilar artery stenosis on three-dimensional (3D) HRMRI.

**Methods:**

Forty-six consecutive patients with symptomatic basilar artery atherosclerotic stenosis on MRA (70–99%) were enrolled. The remodeling index (RI) was the ratio of vessel area at the maximal-lumen-narrowing (MLN) site to reference vessel area. RI ≥ 1.05 was defined as positive remodeling (PR), RI ≤ 0.95 as negative remodeling (NR), and 0.95 < RI < 1.05 as intermediate remodeling (IR). The remodeling patterns were divided into two groups (PR and non-PR [NR and IR]). The cross-sectional and longitudinal distribution of BA plaques were evaluated.

**Results:**

Two patients were excluded because of poor-quality images. Images of 44 patients were available for measurements. PR was found in 23 (52.3%) patients, and non-PR in 21 (47.7%) patients. At the MLN sites, vessel area, wall area, plaque size and percentage of plaque burden of PR group were significantly greater than non-PR group (*p* < .001). Most plaques (90.9%) of the 44 patients were located at the dorsal, left and right walls. For the longitudinal distribution of plaque, 8 (18.2%) and 36 (81.8%) plaques were located in BA proximal and distal to AICA, respectively. Most plaques (68.2%) were eccentrically distributed.

**Conclusions:**

3D HRMRI with postprocessing multiple planar reconstruction is able to evaluate the remodeling pattern and plaque distribution of basilar artery atherosclerotic stenosis, which might be used to guide intracranial intervention.

## Background

Intracranial arterial atherosclerotic stenosis (ICAS) is an important and common cause of ischemic stroke in the world [1]. Patients with intracranial arterial severe stenosis (≥ 70%) face high risk of stroke recurrence, despite best medical treatment [2]. Intracranial percutaneous transluminal angioplasty and stenting (PTAS) has been increasingly used in clinical practice for medically refractory patients [3]. Neurointerventionists generally enrolled symptomatic patients for PTAS based on stenosis severity. However, intracranial arteries have unique vessel wall characteristics (with plenty of penetrating arteries, exhibiting a thin media and adventitia, lacking external elastic lamina), which are different from extracranial arteries [4, 5]. PTAS for ICAS is associated with high rates of periprocedural complications including ischemic and hemorrhagic stroke [6]. The disappointing results of two major randomized controlled trials, the Stenting and Aggressive Medical Management for Preventing Recurrent Stroke in Intracranial Arterial Stenosis (SAMMPRIS) trial and the Vitesse Intracranial Stent Study for Ischemic Stroke Therapy (VISSIT) trial [7, 8], reminded us that lesion characteristics may be one of the important factors affecting the incidence of periprocedural complications.

Previous studies [9–12] showed that morphological characteristics, including pre- existing remodeling and plaque location, were closely related to periprocedural complications. Coronary intervention study found that negative remodeling (NR) was associated with high incidence of in-hospital complications, including post-interventional dissection; in contrast, the rate of major adverse cardiac events in patients with positive remodeling (PR) was higher [9]. In addition, plaque around the branch vessel was associated with branch occlusion following stent placement, which may be due to “snow-plowing” effect (pushing the plaque into branch or perforating arteries in the process of angioplasty or stenting) [10]. Similar periprocedural complications had been shown in the treatment of ICAS with PTAS [7, 8, 11]. Moreover, eccentric lesions are vulnerable to rupture and may lead to distal embolization [12].

Imaging modalities such as digital subtract angiography (DSA), computer tomography angiography (CTA) and magnetic resonance angiography (MRA) mainly show the luminal stenosis and could not provide high quality images for evaluating the morphology of atherosclerotic arterial wall [13]. High-resolution magnetic resonance imaging (HRMRI) has emerged as a valuable tool for evaluating intracranial vessel wall, which can give information about remodeling patterns, plaque location and components [14, 15]. Most of previous studies were performed with 2D black blood technique, in which cross-sectional images perpendicular to the artery long axis may not be acquired for angled lesions or tortuous intracranial artery [15]. Recently, a 3D black blood technique, volumetric isotropic turbo spin echo acquisition (VISTA), is beginning to be used in clinical research [16]. In our study, we aimed to investigate artery remodeling patterns and plaque distribution in symptomatic patients with severe basilar artery (BA) stenosis on 3.0 T MRI scanner.

## Methods

This cross-sectional study was approved by the hospital ethics committees. Each patient signed a written informed consent before examination. All patients fulfilled the following criteria: 1) ischemic stroke or transient ischemic attack(TIA) in the BA territory within 90 days; 2) 70–99% BA stenosis on MRA; and 3) 2 or more vascular risk factors including hypertension, diabetes mellitus, hyperlipidemia, obesity and smoking. Patients were excluded, if having the following conditions: 1) MRI contraindications, for instance, ferromagnetic implants or claustrophobia; 2) non-atherosclerotic vascular disease, such as vasculitis, dissection or moyamoya disease; 3) vertebral artery stenosis (≥ 50%); 4) evidence of cardiogenic cerebral embolism.

Patients were consecutively enrolled in this study from September 2014 to February 2018 if diagnosed as having symptomatic atherosclerotic BA stenosis in the neurology department of our hospital.

### MRI protocol

HRMRI was performed with a 3.0 T MRI scanner (Ingenia; Philips Healthcare, Best, The Nederlands) with a 15-channel phased-array coil. Time of flight (TOF) MRA scan was mainly used to find the location and degree of BA stenosis. 3D VISTA images were obtained by axial plane scanning of the major intracranial arteries with the following parameters: TR/TE = 1300/36 ms, FOV = 140 × 200 × 105 mm^3^, matrix = 280 × 332 × 210, NEX = 2. Acquisition voxel volume was 0.5 × 0.6 × 0.5 mm^3^, and reconstruction voxel volume was 0.5 × 0.5 × 0.5 mm^3^. The axial plane images were automatically constructed with a slice thickness of 0.5 mm. 3D VISTA scan time was about 5 min.

### Measurement and calculation

Two readers evaluated the image quality by using a 4-point scale (1 = poor, 2 = adequate, 3 = good and 4 = excellent) [17]. The two readers had more than 5 year’s experience of reading brain MRI by visual inspection and were blind to the clinical data. The satisfactory images (image quality ≥2)were analyzed using software from the IntelliSpace Portal V6 workstation (Philips Medical Systems, The Netherlands). All the cross-sectional images must be perpendicular to the BA long axis after multiple planar reconstruction (MPR) on the workstation. The images zoomed to 400%, quantitative measurement was performed on the 3D VISTA images. The maximal-lumen-narrowing (MLN) site is the plane where the BA stenosis was severest, and the two reference sites were selected at the point where the vessel walls were parallel immediately proximal and distal to the maximal stenosis. The three special sites were selected for measurement, which was carried out by the first reader.

The vessel area (VA) was traced manually by using the vessel-cerebrospinal fluid interface, and the lumen area (LA) was traced by using the blood-intima interface. Wall area (WA) was defined as VA - LA. The reference VA and LA were average of the distal and proximal reference VA and LA, because of the vessel tapering. Plaque size (PS) was calculated by WA _MLN_ - WA _reference_. Percentage of plaque burden was defined as (PS / VA _MLN_) × 100%. The degree of stenosis was calculated with the following formula: Degree of stenosis = (1 - LA _MLN_ / LA _reference_) × 100% [15, 18]. At last, the remodeling index (RI) was the ratio of VA _MLN_ to VA _reference_. RI ≥ 1.05 was defined as positive remodeling (PR), RI ≤ 0.95 as negative remodeling (NR), and 0.95 < RI < 1.05 as intermediate remodeling (IR). The remodeling patterns were divided into two groups (PR and non-PR [NR and IR]).

Measurements of the wall thickness (WT) were performed by the same reader as above. The eccentricity index was the ratio of (maximal WT - minimal WT) to maximal WT at the MLN site [19]. If the maximal WT was two times larger than the minimal WT, the plaque was considered to be eccentric [20]. The cross-sectional image at the MLN site was divided into four quadrants, namely the right, left, dorsal, and ventral quadrants. If a plaque was distributed on more than 1 quadrant, the quadrant with maximal WT was chosen as the location of plaque [15]. The distribution of plaques in longitudinal direction was also evaluated by dividing BA into two segments, BA proximal and distal to the anterior inferior cerebellar artery (AICA).

To estimate reliability and agreement of measurements, VA and LA of the initial 10 patients at MLN and reference sites were remeasured separately by the 2 readers 2 months later.

### Statistical methods

Most data were analyzed with the Statistical Package for the Social Sciences, Version 17.0 (SPSS, Chicago, Illinois). The intraclass correlation coefficient (ICC) was used to test intraobserver or interobserver variability for the measurements of VA and LA at the three sites. Categorical data was listed as percentage and compared by χ2 test or Fisher’s exact test. Continuous variables were expressed as means ± SD and compared by Student T test. If continuous variables were not normally distributed, it was expressed as an interquartile range and compared by Mann-Whitney U test. *p* value < 0.05 was considered to be statistically significant. The Bland-Altman method was used to test intraobserver or interobserver agreement with MedCalc, Version 18.10.

## Results

### Patients

In this study, 46 consecutive patients were enrolled, in which 2 patients were excluded as a result of poor-quality images. Forty-four patients (32 men and 12 women) who had satisfactory image quality were finally analyzed. The mean age of all patients was 64.3 ± 10.4 years. The mean days from the qualifying events (ischemic stroke or TIA) to HRMRI was 18.0 ± 11.1 days. Baseline characteristics of PR and non-PR patients including age, sex, and various vascular risk factors are illustrated in Table 1, which showed no significant difference between the two groups.

**Table 1:** Baseline characteristics between positive remodeling (PR) and negative remodeling (NR) groups

Characteristics	All (*n* = 44)	PR Group (*n* = 23)	Non-PR Group (*n* = 21)	*p* Value
Age, year, mean (SD)	64.3 (10.4)	62.6 (11.8)	66.2 (8.5)	.251
Men, n (%)	32 (72.7)	20 (71.4)	12 (75.0)	.388
Hypertension, n (%)	41 (93.2)	26 (92.9)	15 (93.8)	1.000
Diabetes mellitus, n (%)	22 (50.0)	13 (46.4)	9 (56.3)	.131
Hyperlipidemia, n (%)	31 (70.5)	21 (75.0)	10 (62.5)	.892
Obesity, n (%)	8 (18.2)	6 (21.4)	2 (12.5)	.302
Smoking, n (%)	18 (40.9)	13 (46.4)	5 (31.3)	.112
Stroke as qualifying event, n (%)	37 (84.1)	25 (89.3)	12 (75.0)	.896
Three or more risk factors, n (%)	30 (68.2)	19 (67.9)	11 (68.8)	.659
NIHSS scores at admission, n, median (interquartile range)	1.0 (0.0–4.0)	1.0 (0.0–4.0)	2.0 (0.0–5.5)	.227
Time from qualifying event to HRMRI, days, median (interquartile range)	14.5 (12.0–20.8)	15.0 (9.0–18.0)	14.0 (12.0–25.0)	.284

*NIHSS* National Institutes of Health Stroke Scale

### Measurement and calculation

**Fig. 1:**
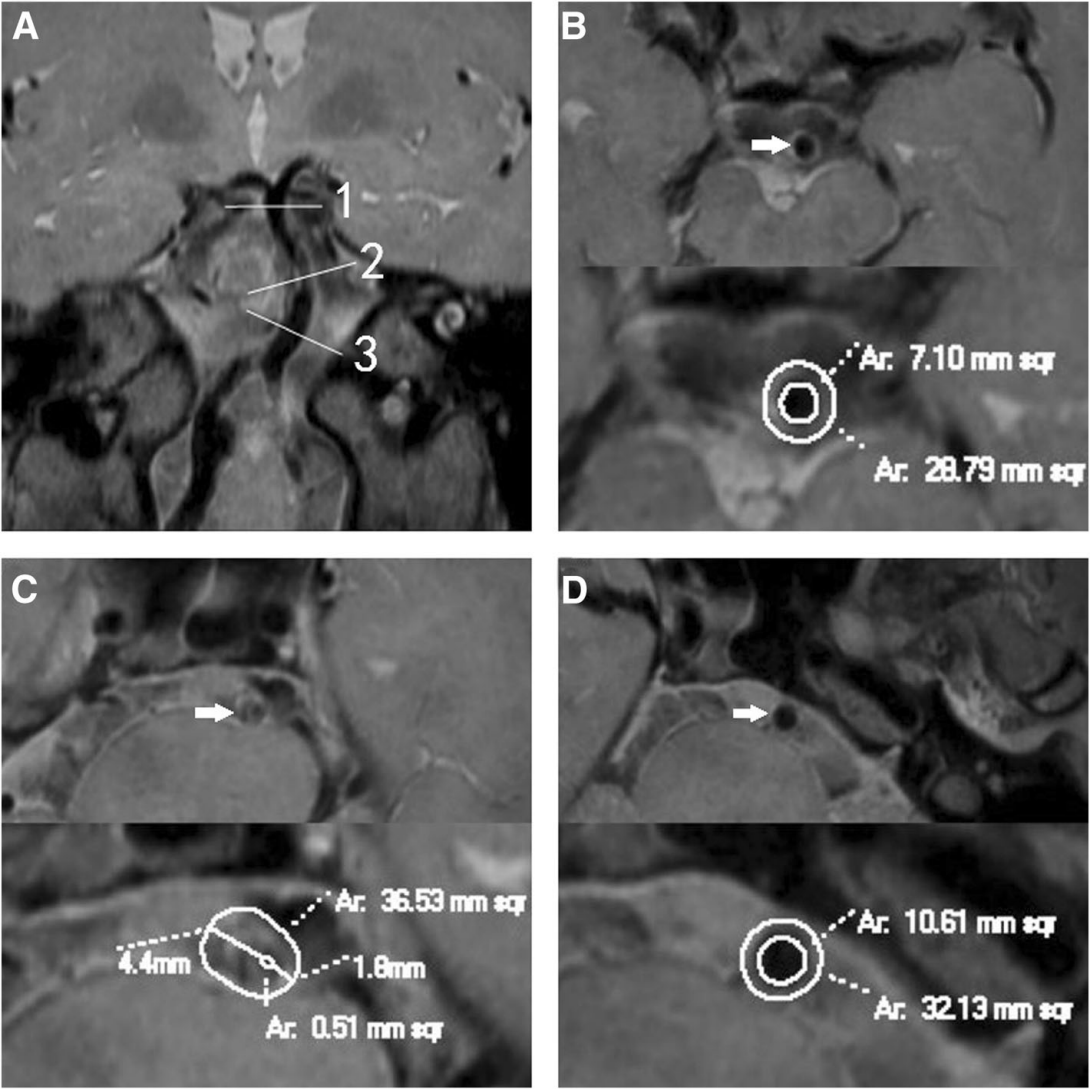
Positive remodeling. A patient (61–65 years) with hypertension and smoking history presented with dizziness for 2 months. Coronal reconstruction image **a** revealed the plaque in BA distal to AICA. The line 1 represents the distal reference site; line 2, maximal-lumen-narrowing (MLN) site; line 3, proximal reference site. Cross-sectional images at the distal, MLN and proximal sites were shown in Figs. **b**, **c** and **d** respectively, as shown by the arrow. The vessel area (VA) is 36.53 mm^2^ at MLN site, 28.79 mm^2^ at distal site, and 32.13 mm^2^ at proximal site. The reference VA is 30.46 mm^2^. The remodeling index (RI) is 1.20 (RI ≥ 1.05, defined as positive remodeling). The lumen area (LA) is 0.51 mm^2^ at MLN site, 7.10 mm^2^ at distal site, and 10.61 mm^2^ at proximal site. The reference LA is 8.86 mm^2^. The wall area (WA) is 36.02 mm^2^ at MLN site, and 21.6mm^2^ at reference site. So the plaque size is 14.42 mm^2^. The maximal wall thickness is 4.4 mm, and the minimal wall thickness is 1.8 mm. The eccentricity index is 0.59, and percentage of plaque burden is 39.5%. The plaque was distributed eccentrically and predominantly located at the right wall

**Fig. 2:**
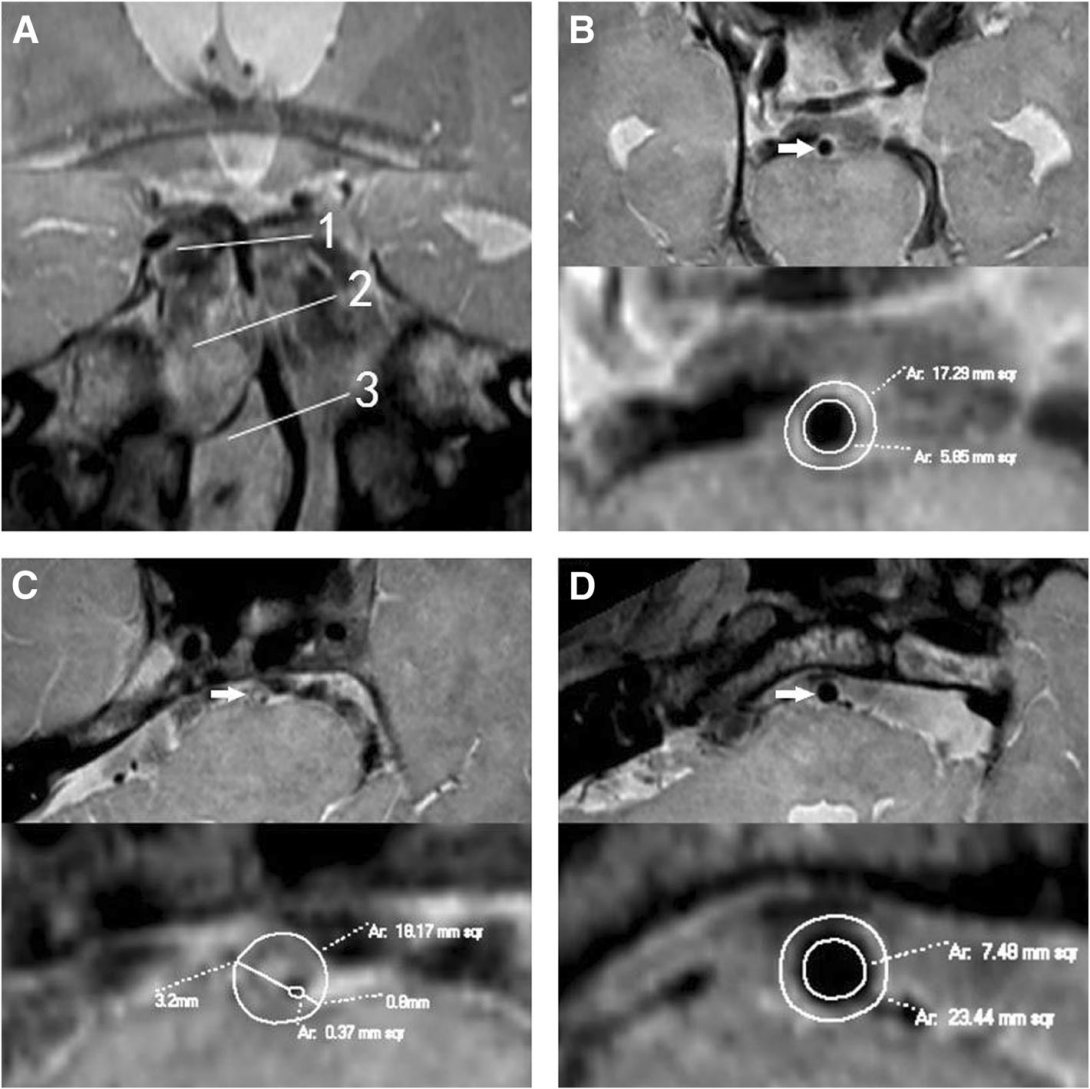
Negative remodeling. A patient (56–60 years) with hypertension, diabetes mellitus and hyperlipidemia presented with left limb weakness for 4 days. Coronal reconstruction image **a** revealed the plaque in BA distal to AICA. The line 1 represents the distal reference site; line 2, maximal-lumen-narrowing (MLN) site; line 3, proximal reference site. Cross-sectional images at the distal, MLN and proximal sites were shown in Figs. **b**, **c** and **d** respectively, as shown by the arrow. The plaque was located in BA distal to AICA. The VA is 18.17 mm^2^ at MLN site, 17.29 mm^2^ at distal site, and 23.44 mm^2^ at proximal site. The reference VA is 20.37 mm^2^. The remodeling index (RI) at the MLN site was 0.89 (RI ≤ 0.95, defined as negative remodeling). The LA is 0.37 mm^2^ at MLN site, 5.85 mm^2^ at distal site, and 7.48 mm^2^ at proximal site. The reference LA is 6.67 mm^2^. The WA is 17.8 mm^2^ at MLN site, and 13.7 mm^2^ at reference site. So the plaque size is 4.1 mm^2^. The maximal wall thickness is 3.2 mm, and the minimal wall thickness is 0.8 mm. The eccentricity index is 0.75, and percentage of plaque burden is 22.6%. Compared with the case above (Fig. 1), this one had smaller VA, WA, plaque size and percentage of plaque burden

PR was found in 23 (52.3%) patients (Fig. 1), and non-PR in 21 (47.7%) patients (Fig. 2). A comparison of the morphologic measurements was shown in Table 2. RI was 1.2 ± 0.1 in PR group and 0.9 ± 0.1 in non-PR group (*p* < .001). At the reference sites, no statistical difference was observed in VA, WA, LA and maximal WT between the two groups. At the MLN sites, PR group had much greater VA, WA, PS and percentage of plaque burden than non-PR group, but the two groups showed no difference in LA. At the same time, maximal and minimal WT of PR group were greater than non-PR group at the MLN sites. Non-PR group had much greater eccentricity index than PR group. Nevertheless, there was no difference in degree of stenosis between the two groups.

**Table 2:** Wall characteristics between positive remodeling (PR) and negative remodeling (NR) groups

Variables(mean ± sd)	All (*n* = 44)	PR Group (*n* = 23)	Non-PR Group (*n* = 21)	*p* value
At the MLN site				
VA (mm^2^)	25.6 (6.4)	29.1 (6.2)	21.8 (4.3)	.000
LA (mm^2^)	0.8 (0.7)	0.8 (0.7)	0.8 (0.7)	.971
WA (mm^2^)	24.8 (6.4)	28.3 (6.1)	21.0 (4.3)	.000
Plaque size (mm^2^)	8.1 (4.2)	11.0 (3.5)	5.0 (2.3)	.000
Percentage of plaque burden (%)	30.3 (11.2)	37.3 (7.4)	22.5 (9.3)	.000
Degree of stenosis (%)	88.9 (9.2)	89.2 (8.6)	88.6 (10.0)	.853
Maximal WT (mm)	3.4 (0.6)	3.6 (0.6)	3.2 (0.5)	.029
Minimal WT (mm)	1.5 (0.5)	1.7 (0.4)	1.2 (0.4)	.000
Eccentricity index	55.8 (16.5)	50.9 (14.4)	61.2 (17.3)	.036
Remodeling index	1.1 (0.2)	1.2 (0.1)	0.9 (0.1)	.000
At the reference site				
VA (mm^2^)	23.8 (5.1)	24.3 (5.4)	23.3 (5.0)	.510
LA (mm^2^)	7.1 (1.9)	7.0 (1.7)	7.2 (2.1)	.684
WA (mm^2^)	16.7 (3.8)	17.3 (3.9)	16.0 (3.7)	.276
Maximal WT (mm)	1.5 (0.3)	1.5 (0.3)	1.4 (0.3)	.171

*VA* vessel area, *LA* lumen area, *WA* wall area, *WT* wall thickness, *MLN* maximal lumen narrowing

### Location and distribution of plaques

Plaques were presented at the ventral wall in 4 (9.1%) patients, the dorsal wall in 13 (29.5%) patients, the left wall in 13 (29.5%) patients, and the right wall in 14 (31.9%) patients (Fig. 1). For the longitudinal distribution of plaque, 8 (18.2%) and 36 (81.8%) plaques were located in BA proximal and distal to AICA, respectively. Most plaques (90.9%) of the 44 patients were located at the dorsal, left and right walls. Most plaques (68.2%) were eccentrically distributed.

### Reliability and agreement of measurements

The intraobserver reliability was high for measurements of VA (at MLN site, ICC = 0.962, 95% CI 0.882–0.988; at proximal reference site, ICC = 0.968, 95% CI 0.899–0.990; at distal reference site, ICC = 0.977, 95% CI 0.927–0.993) and LA (at MLN site, ICC = 0.840, 95% CI 0.556–0.948; at proximal reference site, ICC = 0.924, 95% CI 0.771–0.976; at distal reference site, ICC = 0.904, 95% CI 0.715–0.970). The interobserver reliability was also high for measurements of VA (at MLN site, ICC = 0.898, 95% CI 0.700–0.968; at proximal reference site, ICC = 0.968, 95% CI 0.899–0.990; at distal reference site, ICC = 0.910, 95% CI 0.734–0.972) and LA (at MLN site, ICC = 0.818, 95% CI 0.507–0.941; at proximal reference site, ICC = 0.920, 95% CI 0.759–0.975; at distal reference site, ICC = 0.807, 95% CI 0.481–0.937).

**Fig. 3:**
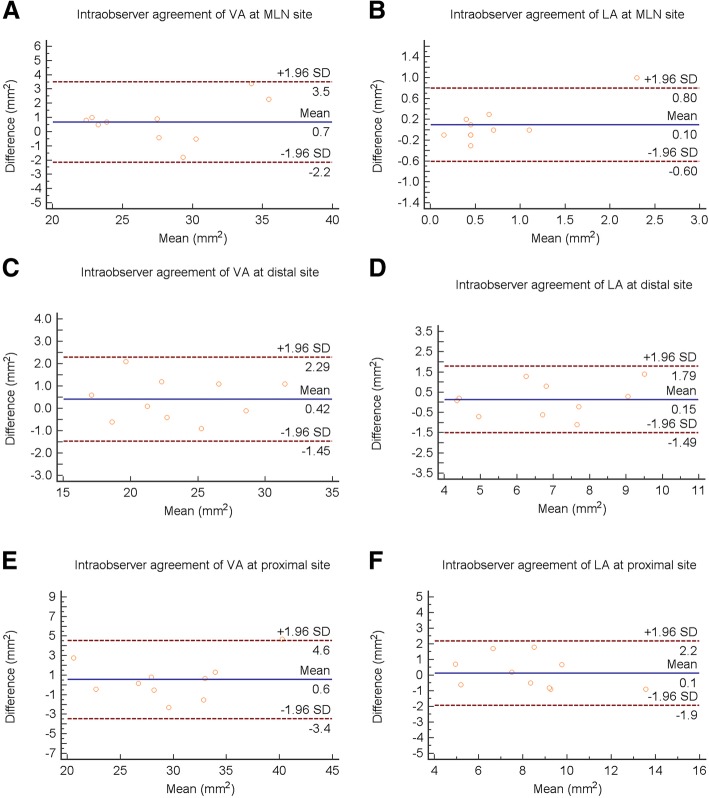
Bland-Altman plots of intraobserver VA and LA measurements at the MLN (**a**, **b**), distal (**c**, **d**) and proximal (**e**, **f**) sites

**Fig. 4:**
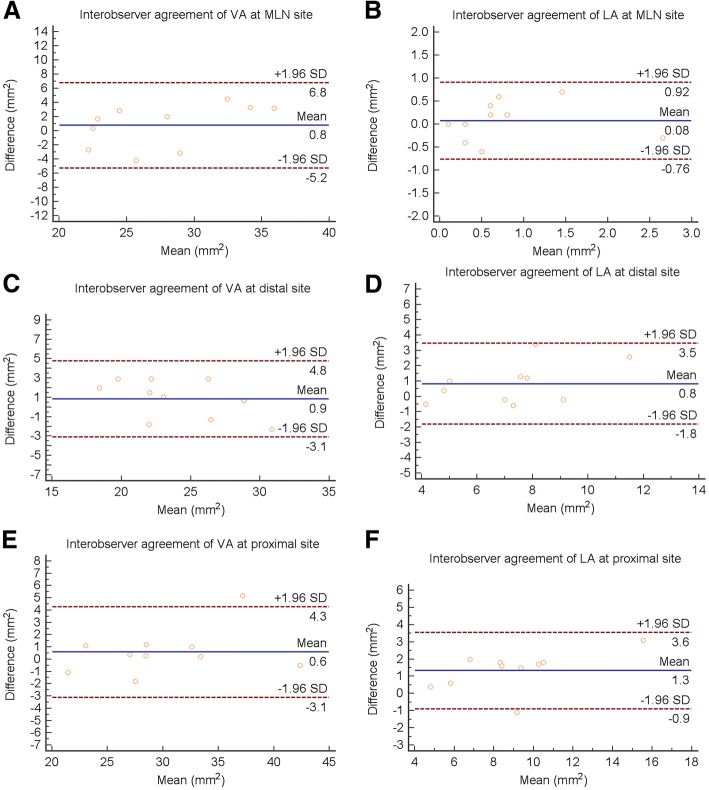
Bland-Altman plots of interobserver VA and LA measurements at the MLN (**a**, **b**), distal (**c**, **d**) and proximal (**e**, **f**) sites

The Bland-Altman plots showed high agreement in intraobserver (Fig. 3) and interobserver (Fig. 4) measurement.

## Discussion

In our study, HRMRI with 3D VISTA can cover major intracranial arteries in a clinical acceptable scanning time. After MPR of 3D images, we can observe intracranial plaques in any planes with high spatial resolution and acquire cross-sectional images at the MLN, distal and proximal reference sites, which were perpendicular to the BA long axis. 3D technique is able to avoid obliqueness artifacts and more suitable for intracranial artery examination than 2D scan which may overestimate the true wall thickness and vessel area [21].

Most researches show that PR is related to unstable plaques that are vulnerable to rupture, while NR is the process of vessel shrinkage, which is relatively stable [22–24]. During interventional therapy, the repeated crossing with a microwire or stent might be prone to plaque rupture. Coronary intervention study showed PR was associated with high incidence of major adverse cardiac events [9]. The previous study also showed that patients with negative remodeling had a high rate of post-interventional dissection [9]. In the process of PTAS, the vessel size is an important factor affecting prognosis [25]. Usually, the balloon or stent size is selected according to the diameter of proximal reference vessel during DSA. For patients with NR, neuroninterventionists should focus on the reduced vessel diameter in the selection of balloon size. Otherwise, it may lead to selecting oversized balloon or stent and increasing the risk of vessel injury. So, a small-sized balloon may be a good choice for patients with NR. The remodeling pattern is an important factor influencing perioperative complications. The preoperative HRMRI can fully assess artery remodeling and guide intracranial PTAS.

In this study, most plaques were distributed eccentrically and predominantly located at the right, left and dorsal walls, where most of perforating arteries originate [26]. Chen et al. [27] found that the most severe plaques of BA were commonly located at the left and right walls, which was similar to our results. Nevertheless, a previous study showed that the majority of middle cerebral artery plaques were located opposite to the orifice of perforating artery [28], different from our study. In the SAMMPRIS trial, patients with BA stenosis had a significantly higher rate of perforator infarct versus other intracranial arteries [29]. Another study reported that the rate of perforator occlusion after middle cerebral artery stenting was very low [30]. The difference in distribution of intracranial plaques may be the important reason of different incidence of perforator stroke in intracranial arteries. PTAS restores the vascular lumen by compressing atheromatous debris into the orifice of perforating arteries (snow-plowing effect). Another study found perforating artery occlusion was the most common cause of ischemic stroke after BA PTAS [10]. Plaques near the orifice of perforating artery increase the risk of perforator infarct [31]. So, for such patients, a small-sized balloon had been suggested to dilate BA stenosis before placing a stent [11]. Eccentric plaques are vulnerable and prone to distal embolism [12]. Vessel dissection or perforation is easy to occur with eccentric lesions during stenting [12]. HRMRI could identify the location of eccentric plaques and may distinguish the true and false lumen, which is helpful to avoid iatrogenic dissection [11]. For patients with severe basilar artery stenosis, PTAS is not a good choice if the eccentric plaques are distributed at bilateral walls. HRMRI’s findings are helpful to select the right patient for treatment, and choose the appropriate stent and balloon.

There are some limitations in our study. First, the sample size was relatively small, and there was no comparative study of pathology. Second, the patient’s clinical follow-up has been ongoing by neurologists, not yet completed. Follow-up information was not included in this cross-sectional study. Third, the results were not applicable to all patients, because our study only included patients with BA stenosis ≥70%.

## Conclusions

After multiple planar reconstruction of 3D VISTA image, high-quality cross-sectional image is able to evaluate the remodeling pattern and plaque distribution of basilar artery atherosclerotic stenosis, which might be used to guide intracranial PTAS.

## Abbreviations

AICA: Anterior inferior cerebellar artery
BA: Basilar artery
HRMRI: High-resolution magnetic resonance imaging
ICAS: Intracranial arterial atherosclerotic stenosis
ICC: Intraclass correlation coefficient
LA: Lumen area
MLN: Maximal lumen narrowing
MPR: Multiple planar reconstruction
NIHSS: National Institutes of Health Stroke Scale
NR: Negative remodeling
PR: Positive remodeling
PS: Plaque size
PTAS: Percutaneous transluminal angioplasty and stenting
RI: Remodeling index
SAMMPRIS: The Stenting and Aggressive Medical Management for Preventing Recurrent Stroke in Intracranial Arterial Stenosis
TIA: Transient ischemic attack
VA: Vessel area
VISTA: Volumetric isotropic turbo spin echo acquisition
WA: Wall area
WT: Wall thickness
